# Malaria vector bionomics in Taita-Taveta County, coastal Kenya

**DOI:** 10.1186/s13071-022-05527-w

**Published:** 2022-11-16

**Authors:** Jonathan Karisa, Kelly Ominde, Simon Muriu, Vanessa Munyao, Kioko Mwikali, Lawrence Babu, Zedekiah Ondieki, Brian Bartilol, Mercy Tuwei, Caroline Wanjiku, Marta Maia, Janet Midega, Martin Rono, Norbert Peshu, Charles Mbogo, Joseph M. Mwangangi

**Affiliations:** 1 KEMRI-Wellcome Trust Research Programme, Kilifi, Kenya; 2 KEMRI-Center for Geographic Medicine Research Coast, Kilifi, Kenya; 3 Population Health Unit, KEMRI-Wellcome Trust Research Programme, Nairobi, Kenya; 4 Department of Biological and Public Health, Pwani University, Kilifi, Kenya; 5 Pwani University Bioscience Research Centre, Pwani University, Kilifi, Kenya; 6 Centre for Global Health and Tropical Medicine, University of Oxford, Oxford, UK

**Keywords:** Malaria, *Anopheles*, Sporozoite, Blood meal, Outdoor transmission, Kenya, Taveta

## Abstract

**Background:**

Estimation of the composition and densities of mosquito species populations is crucial for monitoring the epidemiology of mosquito-borne diseases and provide information on local vectors to public health officials and policy-makers. The aim of this study was to evaluate malaria vector bionomics in ecologically distinct sites in Taita-Taveta County, Kenya.

**Methods:**

Adult mosquitoes were collected using backpack aspirators and paired indoor/outdoor CDC light traps in 10 randomly selected households in six villages with distinct ecologies over a study period of 3 years. All *Anopheles* mosquitoes were morphotyped, and sibling species of *Anopheles gambiae* sensu lato (*An. gambiae* s.l.) were identified and separated by PCR analysis of extracted ribosomal DNA. All female anophelines were tested for sporozoite infectivity, with engorged females screened for blood-meal sources using the enzyme-linked immunosorbent assay technique. A subsample of those testing positive and those testing negative for* Plasmodium* in the ELISA were subjected to PCR assay.

**Results:**

A total of eight different *Anopheles* species were collected both indoors and outdoors. *Anopheles gambiae* s.l. (82.6%, *n* = 5252) was the predominant species sensu lato, followed by *Anopheles coustani* sensu lato (*An. coustani* s.l.; (10.5%, *n* = 666) and *Anopheles funestus* sensu lato (*An. funestus* s.l.; 5.6%, *n* = 357). A subset of 683 mosquito samples representing *An. gambiae* s.l. (*n* = 580, approx. 11.0%) and *An. funestus* s.l. (*n* = 103, approx. 28.9%) were identified by molecular diagnostic assays into sibling species. The *An. gambiae* s.l. complex was composed of *Anopheles arabiensis* (62.5%, *n* = 363/580), *An. gambiae* sensu stricto (*An. gambiae* s.s.; 0.7%, *n* = 4/580), *Anopheles merus* (0.7%, *n* = 4/580) and *Anopheles quadriannulatus* (0.2%, *n* = 1/580), with the remaining samples (35.5%, *n* = 206/580) unamplified. *Anopheles funestus* s.l. was composed of *An. rivulorum* (14.6%, *n* = 15/103) and *An. leesoni* (11.6%, *n* = 12/103); the remaining samples were unamplified (73.8%, *n* = 76/103). A total of 981 samples were subjected to PCR analysis for malaria parasite detection; of these 16 (1.6%) were confirmed to be positive for *Plasmodium falciparum*. The overall human blood index was 0.13 (32/238).

**Conclusions:**

*Anopheles gambiae, An. funestus* and *An. coustani* are key malaria vectors in the Taveta region of Kenya, showing concurrent indoor and outdoor transmission. All of the vectors tested showed a higher propensity for bovine and goat blood than for human blood.

**Graphical Abstract:**

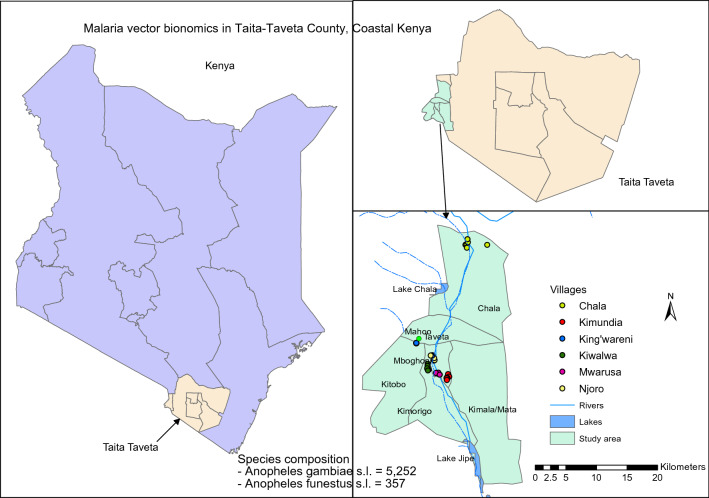

## Background

Despite significant advances in the past two decades nearly half of the global population continues to be at risk for malaria transmission. According to WHO, over 200 million cases and 409,000 deaths were reported in 2020 [1], with the most burdened countries located in WHO’s African Region. A few African countries, such as Algeria and Morocco, have been certified as malaria-free, while others, such as Zimbabwe, Botswana, Swaziland and South Africa, are listed among those on the verge of malaria elimination. However, high-burden African countries, such as Nigeria, Democratic Republic of Congo (DRC), Mozambique, Ivory Coast, Tanzania and Uganda, reported an increase in malaria cases [1, 2], with > 90% of the malaria cases reported in 2020 in these countries [1]. The elimination and reduction of malaria cases has been attributed to widespread use of interventions, such as prompt diagnosis, treatment and vector control strategies [2, 3].

Malaria is endemic in Kenya, with the majority of affected populations living in the western and coastal areas of Kenya. Epidemiology is governed by rainfall patterns, temperature and altitude, and is driven by distinct vectors that are dependent on specific ecological conditions. Vector control is an essential component and an important cornerstone of malaria prevention, control and elimination strategies by effectively reducing vector-human contacts and thereby reducing disease transmission [4, 5]. Malaria reduction in coastal Kenya has been mainly due to coverage and widespread use of long-lasting insecticide-treated nets (LLINs), while in western Kenya the combination of LLINs and indoor residual spraying have been the drivers of malaria reduction [4, 6–9]. It has been noted that this reduction in disease burden was accompanied by changes in the vector populations, potentially due to selective pressure, with the changes including diversity, role as vectors and behaviour. Species with greater behavioural plasticity were favoured, particularly those that bite both outdoors and outside regular sleeping hours as well as biting both humans and peri-domestic animals [10–14].

In Kenya, the composition and distribution of malaria vector species are highly heterogenous. *Anopheles funestus* is described an emerging main malaria vector in the highland regions of Kenya. However, in the low-altitude arid and semi-arid areas of the coastal region, *Anopheles arabiensis* is the predominant vector [15]. In some areas of coastal and western Kenya, *Anopheles coustani*, a secondary vector, is now becoming a major vector and contributing substantially to malaria transmission [10, 16]. In much of coastal Kenya, *An. arabiensis* has replaced *Anopheles gambiae* sensu stricto (*An. gambiae* s.s), and *An. funestus* sensu stricto (s.s.) has become the dominant malaria vector [15]. Moreover, several secondary zoophillic, exophagic, and exophilic vectors may also play a role in transmission, including *An. coustani* sensu lato (s.l.) *Anopheles pharoensis* sensu lato (*An. pharoensis *s.l.), *Anopheles rivulorum, Anopheles parensis, Anopheles merus* and *Anopheles quadriannulatus* [10, 14, 16–19]. Most vectors that evade control measures also present with diverse trophic preferences, including humans but also cattle, goats, chicken, dogs and pigs [15, 20–22], thus allowing vector populations to persist and increase in number. The changes in species composition, increasing role of secondary vectors, feeding patterns, heterogeneity in transmission focal points and geographic expansion of vectors has implications for the control of malaria and disease elimination efforts. Furthermore, the geographic expansion of the invasive urban malaria vector *Anopheles stephensi* into the Horn of Africa calls for improved surveillance strategies not only in rural areas but also in urban environments [23].

Surveillance and control strategies require prior knowledge on the vector bionomics, species composition, biting behaviours and sporozoite rates of local vector species that transmit malaria. Hence, a 3-year entomological surveillance was undertaken in Taita-Taveta County, Kenya to gain an understanding of malaria transmission dynamics and gather information on local vector species diversity, trophic preferences and plasmodial infection status.

## Methods

### Study site

The study was carried out in the subcounty of Taveta, Taita-Taveta County (3°24′00″ S, 37°41′00″ E), coastal Kenya. Taveta subcounty is located about 109 km west of Voi town, off the Nairobi-Mombasa road, and is mainly inhabited by members of the Taita and Taveta ethnic groups. The primary occupations of the residents of this subcounty are mixed farming, livestock, trade/business and casual wage labour. The area is a plateau that generally slopes towards the south and is at 752 m a.s.l. The mean annual rainfall ranges between 200 and 1200 mm, with long rains between March and May and the long dry season occurring between June and October. Mean annual temperatures range between 21 °C and 31 °C. Irrigated agricultural activities are the main source of livelihood, with water for irrigation obtained from the Tsavo, Lumi, Njoro and Kitobo rivers, as well as from spring water at the foot of Mount Kilimanjaro.

**Fig. 1:**
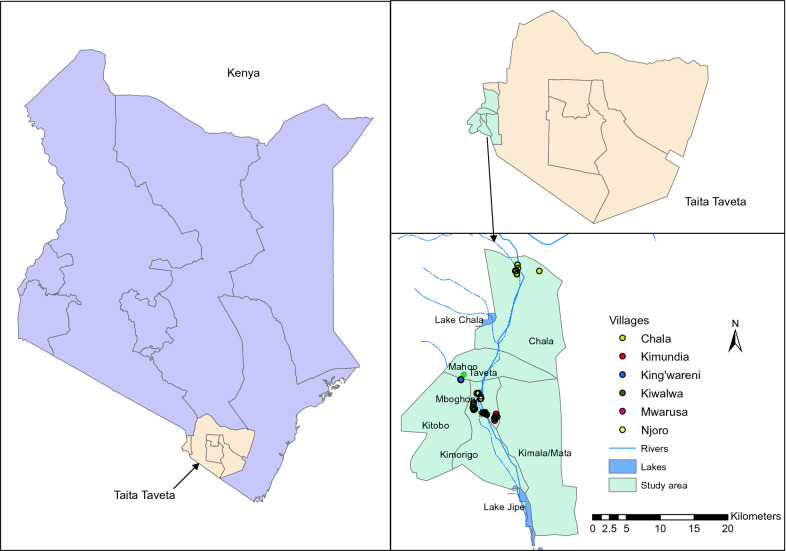
Maps showing the location of the study sites where entomological sampling was conducted between 2016 and 2018

Taita-Taveta County is malaria endemic [8, 24], with perennial transmission at the end of the long rainy season [25–27]. Prevalence rates are rising, as evidenced from clinical malaria data reported by the Kenyan National Malaria Control Programs (NMCP) [24]. In 2011, the annual entomological inoculation rate in Taita-Taveta County was about 31.13 infective bites per person per year [10]. In the present study, six villages representing different ecological and hydrological (irrigated vs non-irrigated) set-ups were targeted for mosquito surveillance: Kiwalwa, Mwarusa, Njoro, Kimundia, Kingwareni and Chala villages (Fig. 1).

### Entomological sampling

Adult mosquitoes were collected using the Centers for Disease Control and Prevention (CDC) Backpack Aspirator and paired indoor/outdoor CDC light traps as previously described [10]. Ten houses were randomly selected in each village for mosquito collection. Paired CDC light mosquito trapping was done, with one trap set indoors and the second trap located outdoors (a few meters from an animal enclosure), from 1800 hours to 0600 hours. Indoor resting mosquitoes were collected from five randomly selected houses in each village using the CDC Backpack Aspirator between 0600 hours and 1000 hours. All samples collected were transported to the field laboratory at Taveta station for sorting and preservation before being transported to the laboratories of the Kenyan Medical Research Institute Wellcome Trust Research Programme (KWTRP) in Kilifi for further processing. Samples were collected in 2016 (November), 2017 (February, March) and 2018 (March, April, October).

### Morphological identification and processing of mosquitoes

Female *Anopheles* mosquitoes were identified to species level using dichotomous morphological keys [28], following which they were separated according to physiological status. The mosquitoes were then dissected into legs and wings, head and thorax, and abdomen (blood fed) compartments, and placed individually in well-labelled sterile 1.5-ml Eppendorf tubes.

### Molecular identification of the main malaria vectors

DNA was extracted from the legs and wings and subsequently used for sibling species identification by PCR as previously described [29], with slight modification. Briefly, 70 µl of 5% Chelex 100 chelating resin was added to the 1.5-ml vial containing the legs and wings of one mosquito, followed by the addition of one steel bead (diameter: 5 mm) and subsequent homogenization using a tissue lyser (1 cycle; frequency: 30 Hz; duration: 1 min) [30]. The homogenate was then incubated at 95 °C for 10 min, followed by centrifugation at 15,000 rpm for 10 min. The supernatant was then transferred into a sterile 1.5-ml vial. To ensure complete removal of the Chelex from the DNA, another centrifugation at 15,000 rpm for 10 min followed. Finally, the supernatant was transferred into a sterile 1.5-ml vial and stored at − 80 °C until further processing. For *An. gambiae* sensu lato (*An. gambiae* s.l.), genomic DNA was amplified using specific primers targeting the intergenic spacer region (IGS2) of the ribosomal DNA [31]. We screened for *An. gambiae* s.s., *An. arabiensis*, *An. merus* and *An. quadriannulatus* [31]. The mosquito genomic DNA for *An. funestus* sensu lato (*An. funestus* s. l.) was amplified using specific primers targeting the internal transcribed region 2 (ITS2) from the 5.8S and 28S coding region flanking the variable ITS2 region [32]. The reaction volume of 15 µl contained 5 µl of 2× GoTaq Green Master Mix (Promega Corp., Madison, WI, USA), 0.5 µl of each primer (concentration: 10 µM), 4.5 µl of nuclease-free water and 3 µl of genomic DNA. The thermocycling conditions were: an initial denaturation at 95 °C for 2 min, followed by 35 cycles of 95 °C for 30 s, 55 °C for 45 s and 72 °C for 30 s, with a final elongation at 72 °C for 10 min. The resulting amplicons were visualized in 1.5% agarose with RedSafe™ Nucleic Acid Staining Solution (magnification: 20,000×) (iNtRON Biotechnology, Sangdaewon-Dong, Gyeonggi-do, South Korea) using a UV photographer (Bio-Rad Laboratories, Hercules, CA, USA).

### *Plasmodium* screening in mosquitoes

All anopheline mosquitoes collected were tested for sporozoite infectivity using a specific *Plasmodium falciparum* sporozoite enzyme-linked immunosorbent assay (ELISA) as previously described [15]. The head and thorax compartment of individual mosquitoes were homogenized in 250 µl of grinding buffer using a sterile pestle. The microtiter plate was first coated with the primary *P. falciparum* capture monoclonal antibodies (mAb) followed by blocking of any unbound sites using blocking buffer. A 50-μl sample of the mosquito homogenate was then put added to wells of the microtiter plate and incubated for 2 h. The plate was then rinsed, followed by the addition of 100 µl anti-sporozoite monoclonal peroxidase-labelled conjugate antibody (secondary mAb). After a second rinsing of the microtiter plate, 100 µl of 2,2ʹ-azino-bis (3-ethylbenzothiazoline-6-sulfonic acid) (ABTS) substrate added to each well. The results were read qualitatively (visually) based on colour change, with a homogenous greenish-blue colour considered to indicate positivity and a clear sample without any colour change considered to indicate negativity. For quality assurance purposes, each microtiter plate was loaded with a positive control (BEI Resources, Manassas, VA, USA) and a negative control (naïve females of *An. gambiae* s.s (obtained from insectary colony) that had never obtained an infectious blood meal). The *P. falciparum* Sporozoite ELISA Reagent Kit (MRA-890) was obtained through BEI Resources, US National Institute of Allergy and Infectious Diseases (NIAID) and US National Institutes of Health (NIH) (both Bethesda, MD, USA), contributed by Robert A. Wirtz (CDC, Atlanta, GA, USA) [15].

A subsample of those samples testing positive and negative, respectively, in the ELISA were analysed by PCR as previously described [33]. Molecular detection by PCR was conducted in 2022, several years after sampling. DNA was extracted from the samples by mixing 100 µl of the remaining homogenate (lysate) with an equal volume of 5% Chelex solution (w/v). The mixture was thoroughly vortexed to ensure complete mixing, followed by incubation at 95 °C for 10 min. This was followed by centrifugation at 15,000 rpm for 10 min. The supernatants were transferred into respective sterile 1.5-ml vials. To ensure complete removal of the Chelex from the DNA, another centrifugation at 15,000 rpm for 10 min was performed. Finally, the supernatants were transferred into respective sterile 1.5-ml vials and stored at − 80 °C until further processing. The obtained DNA was subjected to PCR analysis. The primers used target a subunit of the cytochrome* c* oxidase I gene (*COX1*) located in the plasmodium mitochondrial genome. The set of primers used were COX-IF (forward primer: 5′-AGAACGAACGCTTTTAACGCCTG-3′) and COX-IR (reverse primer: 3′-ACTTAATGGTGGATATAAAGTCCATCCwGT-5′). The reaction volume of 20 µl contained 10 µl of 2× GoTaq Green Master Mix (Promega Corp.), 0.5 µl of each primer (concentration: 10 µM), 5 µl of PCR water (nuclease free) and 4 µl of genomic DNA. The PCR cycling conditions were: an initial denaturation at 95 °C for 2 min, followed by 40 cycles of 95 °C for 30 s, 56 °C for 45 s and 72 °C for 30 s, with a final elongation at 72 °C for 2 min. The resulting amplicons were visualized in a UV photographer after gel electrophoresis (Bio-Rad Laboratories). To quality check if the sample DNA was degraded or not (in the unamplified samples), we performed species identification as described above [31].

### Blood-meal analysis

Blood-meal analyses to determine mosquito feeding preferences were conducted using a previously described protocol [20, 34, 35]. Direct ELISA assay was used to discriminate blood-meal sources by using anti-host (immunoglobulin G) conjugate against humans, bovines, goats and chickens. Briefly, the abdomens of individual mosquitoes were homogenized in 1000 µl of 1× phosphate-buffered saline (PBS) using sterile pestles. A 50-µl sample of the homogenates was coated to the wells of the microtiter plate and incubated at room temperature for 30 min. The contents were then aspirated out, the wells washed and 50 µl of conjugated mAbs was added. Lastly, 100 µl of ABTS substrate was added to the well. Each step was followed by incubation at room temperature as well as rinsing of the plate. The results were read qualitatively (visually) through colour change, with a homogenous greenish-blue colour considered to indicate positivity and a clear sample without colour change considered to indicate negativity, as previously described [20, 36]. The positive controls were serum from human, bovine, goat, and chicken, whereas PBS was used as negative control.

### Data analysis

Data collected were entered into Microsoft Excel (Microsoft Corp., Redmond, WA, USA) and analysed using R studio statistical software version 4.0.2 [37]. The human blood index (HBI), defined as the proportion of mosquitoes that obtained blood from human hosts, was calculated by dividing the number of mosquitoes that obtained their meals from humans (including mixed blood-meal origins) by the total number of blood-fed *Anopheles* mosquitoes analysed. Human biting rate (HBR) by CDC light trap was calculated by dividing the number of female anopheline mosquitoes captures by the number of collection events.

We described discrete (*An. gambiae* and *An. funestus*) and categorical variables by calculating medians and proportions, respectively. Mosquito densities (*An. gambiae* and *An. funestus*) in each location over time was summarized using box and whiskers plot. We fitted a generalized linear model of negative binomial distribution with a log function to determine the association between the outcomes and independent variables (location, roof type, wall type and the presence of eaves). The estimated coefficients and 95% confidence interval in both models were reported, and all predictors with *P* < 0.05 were significantly associated with outcome variables (*An. gambiae* and *An. funestus*). All statistical analyses were performed using Stata version 15.0 (Stata Corp., College Station, TX, USA).

## Results

### Spatial and temporal distribution of malaria vectors

A total of 6361 *Anopheles* mosquitoes were collected comprising of eight *Anopheles* species. Table 1 summarizes the malaria vector species and their distribution in the six villages between 2016 and 2018. *Anopheles gambiae* s.l*.* (82.57%, *n* = 5252) was the predominant species followed by *An. coustani* (10.47%, *n* = 666), *An. funestus* (5.61%, *n* = 357), *Anopheles pretoriensis* (0.41%, *n* = 26)*, An. pharoensis* (0.88%, *n* = 56)*, Anopheles squamosus* (0.0.03%, *n* = 2), *Anopheles maculipalpis* (0.02%, *n* = 1) and *Anopheles moucheti* (0.02%, *n* = 1). Overall, significantly high densities of *Anopheles* mosquitoes were recorded during the wet season (*n* = 5867, 92.2%) compared to the dry season (*n* = 494, 7.8%). *Anopheles gambiae* s.l. accounted for > 96% of mosquitoes collected during the wet season while *An. coustani* accounted for 84.8% (*n* = 565) of mosquitoes collected during the same period. However, *An. funestus* s.l. were most abundant during the dry season (*n* = 187, 52.4%) as opposed to the wet season (*n* = 170, 47.6%) (Table 1). An equilibrium was observed in the densities of anophelines/*Anopheles* captured indoors (*n* = 3388, 53.3%) and outdoors (*n* = 2973, 46.7%). Among the *An. gambiae* and *An. funestus* s.l., a tendency towards endophily was observed. Approximately two-thirds of *An. gambiae* (58.7%) were captured indoors compared to 2171 (41.3%) captured outdoors. A slight majority of *An. funestus* s.l. were collected indoors (53%) compared to indoors (47%). A minor malaria vector, *An. coustani* was recorded to have a significantly higher density outdoors (*n* = 559, 83.9%) than indoors (*n* = 107, 16.1%).

**Table 1:** Spatial and temporal distribution of* Anopheles* mosquito species in Taveta subcounty

Parameters	*An. funestus*	*An. gambiae*	*An. coustani*	*An. maculipalpis*	*An. moucheti*	*An. pharoensis*	*An. pretoriensis*	*An. squamosus*
Subtotal	357 (5.6)	5252 (82.6)	666 (10.5)	1 (0.02)	1 (0.02)	56 (0.9)	26 (0.4)	2 (0.03)
*Season*								
Dry	187 (52.4)	192 (3.66)	101 (15.2)	0 (0.0)	0 (0.0)	2 (3.57)	12 (46.2)	0 (0.0)
Wet	170 (47.6)	5,060 (96.3)	565 (84.8)	1 (10.0)	1 (10.0)	54 (96.4)	14 (53.8)	2 (100)
*Site*								
Chala	3 (0.84)	18 (0.34)	0 (0.00)	1 (10)	0 (0.0)	0 (0.00)	0 (0.0)	0 (0.0)
Kimundia	65 (18.2)	2218 (42.2)	487 (73.1)	0 (0.0)	0 (0.0)	23 (41.1)	0 (0.0)	2 (100.0)
Kingwareni	36 (10.1)	566 (10.8)	32 (4.80)	0 (0.0)	0 (0.0)	3 (5.36)	0 (0.0)	0 (0.0)
Kiwalwa	103 (28.9)	1372 (26.1)	69 (10.4)	0 (0.0)	0 (0.0)	5 (8.9)	0 (0.0)	0 (0.0)
Mwarusa	24 (6.72)	756 (14.4)	34 (5.1)	0 (0.0)	0 (0.0)	20 (35.7)	0 (0.0)	0 (0.0)
Njoro	126 (35.3)	322 (6.13)	44 (6.6)	0 (0.0)	1 (100.0)	5 (8.93)	26 (100.0)	0 (0.0)
*Location*								
Indoors	190 (53.2)	3081 (58.7)	107 (16.1)	1 (100)	0 (0.0)	8 (14.3)	0 (0.00)	1 (50.0)
Outdoor	167 (46.8)	2171 (41.3)	559 (83.9)	0 (0.00)	1 (100.0)	48 (85.7)	26 (100.0)	1 (50.0)
*Year*								
2016	86 (24.1)	553 (10.5)	48 (7.21)	0 (0.00)	0 (0.0)	4 (7.1)	13 (50.0)	0 (0.0)
2017	45 (12.6)	717 (13.7)	145 (21.8)	0 (0.00)	0 (0.0)	2 (3.6)	12 (46.2)	2 (100.0)
2018	226 (63.3)	3982 (75.8)	473 (71.0)	1 (100)	1 (100.0)	50 (89.0)	1 (3.85)	0 (0.0)

Values in table are given as the number (*n*) of collected specimens with the percentage of each species according to season, site, location and year given in parentheses

The highest number of mosquitoes were collected in Kimundia (43.9%, *n* = 2795) followed by Kiwalwa (24.4%, *n* = 1549), Mwarusa (13.1%, n = 834), Kingwareni (10.0%, *n* = 637), Njoro (8.2%, *n* = 524) and Chala (0.35%, *n* = 22). *Anopheles gambiae* s.l. was reported in all the sites in varying densities, with higher numbers in Kimundia (*n* = 2218) followed by Kiwalwa (*n* = 1372), Mwarusa (*n* = 756), Kingwareni (*n* = 566), Njoro (*n* = 322) and Chala (*n* = 18). *Anopheles funestus* was analogously reported in all the sites in varying densities, with higher numbers in Njoro (*n* = 126) followed by Kiwalwa (*n* = 103), Kimundia (*n* = 65), Kingwareni (*n* = 36), Mwarusa (*n* = 24) and Chala (*n* = 3). *Anopheles coustani* (a secondary vector), on the other hand, was reported in all the sites except Chala, and still at varying densities across the different sites, as demonstrated in Table 1. Compared to all other sites, the highest number (*n* = 487) of *An. coustani* samples was collected in Kimundia. *Anopheles pharoensis* was reported in varying densities in all the sites except Chala (Table 1). Other* Anopheles* mosquitoes collected include *An. maculipalpis* (*n* = 1) in Chala, *An. moucheti* (*n* = 1) and *An. pretoriensis* (*n* = 26) in Njoro and *An. squamosus* (*n* = 2) in Kimundia (Table 1). Overall, individuals in Taita-Taveta County received about 16.89 bites per person per night (b/p/n). A significantly majority of these bites were from *An. gambiae* (13.9 b/p/n), with *An. coustani* and *An. funestus* contributing a marginal 2.0 and 1.0 b/p/n, respectively.

### Effect of location and household characteristics on *An. gambiae* and *An. funestus* counts

Multivariate analysis indicated that the covariates roof type and eaves were positively associated with increased *An. gambiae* counts. Interestingly, sampling mosquitoes in outdoor locations was associated with a decrease in *An. gambiae* counts (Table 2). Multivariate analysis also indicated that the covariates/predictors roof type (grass), location (indoor vs outdoor) and eaves were positively associated with increased *An. funestus* counts. The other covariates were negatively associated with *An. funestus* counts (Table 2).

**Table 2:** Multivariate analysis of factors influencing the collection of *An. gambiae* and *An. funestus* in villages of Taita-Taveta County

Independent characteristics	*An. gambiae*			*An. funestus*		
	Estimate	95% Confidence interval	*P-*value	Estimate	95% Confidence interval	*P-*value
*Wall*						
Mud	Reference					
Blocks	− 0.79	− 1.25, − 0.34	< 0.001	− 0.12	− 0.93, − 0.70	0.78
Iron-sheets	− 12.07	− 13.42, − 10.71	< 0.001	− 10.79	− 12.44, − 09.14	< 0.001
Grass	− 13.55	− 15.72, − 11.39	< 0.001	− 11.24	− 13.28, − 09.21	< 0.001
*Roof type*						
Iron sheet	Reference					
Grass	1.25	− 0.46, 2.95	0.15	1.54	0.34, 2.73	0.01
Makuti	0.34	− 0.39, − 1.06	0.36	− 0.14	− 1.68, 1.40	0.86
*Location*						
Indoor	Reference					
Outdoor	− 0.06	− 0.43, 0.32	0.77	0.01	0.44, 0.45	0.97
*Eaves*						
Yes	Reference					
No	0.24	− 0.6, 1.07	0.58	1.05	0.08, 2.03	0.03

### Molecular identification of *An. gambiae* and *An. funestus* s.l.

A subset of 683 mosquito samples representing *An. gambiae* s.l. (*n* = 580, approx. 11.0%) and *An. funestus* (*n* = 103, approx. 28.9%) were identified by molecular diagnostic assays into sibling species. The *An. gambiae* s.l. complex was composed of: *An. arabiensis* (62.5%, *n* = 363/580), *An. gambiae* s.s (0.7%, *n* = 4/580), *An. merus* (0.7%, *n* = 4/580) and *An. quadriannulatus* (0.2%, *n* = 1/580), with other samples (*n* = 35.5%, *n* = 206/580) unamplified. The *An. funestus* s.l. complex was composed of: *An. rivulorum* (14.6%, *n* = 15/103) and *Anopheles leesoni* (11.6%, *n* = 12/103), with all other samples (73.8%, *n* = 76/103) unamplified. The high rate of unamplified samples could be due to DNA degradation as samples were analysed 7 years after collection.

### Malaria transmission foci

A total of 6361 anopheline mosquitoes were tested for *P. falciparum* sporozoites using circumsporozoite-ELISA. *Plasmodium falciparum* transmission intensity varied according to site and location (indoors vs outdoor) (Table 3). *Plasmodium falciparum* sporozoite-infected mosquitoes were collected both indoors and outdoors in the study area (Table 4). *Anopheles gambiae* and *An. coustani* were observed transmitting malaria both indoors and outdoors, while *An. funestus* was only recorded transmitting malaria outdoors. A subsample of 981 samples consisting of those which tested either positive (*n* = 36) or negative (*n* = 945) in the ELISA were subjected to PCR amplification for confirmation. Of the 36 samples that tested positive for *P. falciparum* sporozoites in the ELISA, positivity was confirmed in 16 samples (6 *An. arabiensis*, 6 *An. coustani,* 4 unamplified) by PCR (Table 3). This difference could be attributable to false positivity by ELISA, as has been reported previously [38, 39]. All samples which tested negative for *P. falciparum* sporozoites in the ELISA also tested consistently negative by PCR. Due the limitation of only analysing a subsample for confirmation purposes, no entomological endpoints were calculated. Moreover, sample analysis by PCR was conducted several years after collection, with the possibility that the DNA material in some samples could have degraded.

**Table 3:** Summary of the proportions of *Plasmodium*-positive samples (as an indicator of malaria transmission foci) by season, site and location of mosquito collection in Taveta subcounty

Parameters	ELISA^a^			PCR^b^		
	*An. coustani*	*An. funestus*	*An. gambiae*	*An. coustani*	*An. arabiensis*	Unamplified samples
*Season*						
Dry	3 (20.0)	0 (0.0)	0 (0.0)	1 (16.7)	0 (0.0)	0 (0.0)
Wet	12 (80.0)	1 (100)	30 (100)	5 (83.3)	6 (100)	4 (100)
*Site*						
Kimundia	12 (80.0)	0 (0.00)	10 (33.3)	5 (83.3)	5 (83.3)	0 (0.0)
Kingwareni	0 (0.00)	0 (0.00)	2 (6.67)	0 (0.00)	0 (0.0)	1 (25.0)
Kiwalwa	2 (13.3)	0 (0.00)	11 (36.7)	1 (16.7)	1 (16.7)	1 (25.0)
Mwarusa	1 (6.67)	0 (0.00)	3 (10.0)	0 (0.00)	0 (0.0)	2 (50.0)
Njoro	0 (0.00)	1 (100)	4 (13.3)	0 (0.00)	0 (0.0)	0 (0.0)
*Location*						
Indoors	2 (13.3)	0 (0.00)	24 (80.0)	0 (0.00)	3 (50.0)	4 (100)
Outdoor	13 (86.7)	1 (100)	6 (20.0)	6 (100)	3 (50.0)	0 (0.0)

Values in table are given as the number (*n*) of *Plasmodium*-positive samples with the percentage of positive samples for each species according to season, site and location given in parentheses

*ELISA* Enzyme-linked immunosorbent assay

^a^*Plasmodium* was detected using an ELISA targeting *P. falciparum* circumsporozoite protein

^b^The PCR assay used primers that target a subunit of the cytochrome c oxidase I gene (*COX1*) located in the* Plasmodium* mitochondrial genome. (Parentheses indicate percentages of the plasmodium positivity)

### The feeding preference of key malaria vectors

 The blood-meal sources for the mosquitoes analysed are shown in Table 4. Of the 238 (217 indoors vs 21 outdoors)* Anopheles* mosquitoes tested for blood-meal sources, consisting of *An. gambiae* s.l. (92%, *n* = 219), *An. funestus* s.l. (5.9%, *n* = 14) and *An. coustani* (2.1%, *n* = 5), a significant majority had fed on single hosts, namely bovine (*n* = 80), goat (*n* = 33) and, at the lowest frequency, humans (*n* = 24). Regarding mixed blood-meal sources, the majority of the samples tested showed blood from the following combinations: bovine-goat (*n* = 18), human-goat (*n* = 4), bovine-human (*n* = 3) and, at the lowest frequency, bovine-human-goat (*n* = 1). In the remaining samples (31.4%, *n *= 75) the blood-meal source could not be identified. *Anopheles gambaie* s.l. preferentially fed on bovine (36.4%, *n* = 80), followed by humans (10.9%, *n* = 24) and goats (10.0%, *n* = 22), and 10.0% (*n* = 22) obtained their blood meals from mixed hosts, as demonstrated in Table 4. For the 14 *An. funestus* tested, the majority (57.1%) preferred obtained blood meals from goat, and 21.4% obtained mixed meals from the bovine-goat combination. *Anopheles coustani* showed a higher propensity for feeding on goat (60%, *n* = 3) and bovine (20%, *n* = 1) and mixed meals on bovine-human (20%, *n* = 1). The overall HBI was 0.13, with *An. gambiae* s.l. having the highest HBI (0.129) followed by *An. coustani* (HBI: 0.004) and *An. funestus* (HBI: 0), as none of these was found to have obtained blood from humans (Table 4).

**Table 4:** Proportion of blood-feeding preferences among the mosquitoes collected in the six villages in Taveta subcounty

Species	No.* Anopheles* mosquitoes tested for blood-meal sources	Single host			Mixed feeding				Unidentified
		Human	Bovine	Goat	Bovine–Goat	Boven–Human	Human–Goat	Bovine–Human–Goat	
*An. funestus*	14	0 (0.0)	0 (0.0)	8 (57.1)	3 (21.4)	0 (0.0)	0 (0.0)	0 (0.0)	3 (21.4)
*An. gambiae*	219	24 (11.0)	79 (36.1)	22 (10.0)	15 (6.8)	2 (0.9)	4 (1.8)	1 (0.5)	72 (32.9)
*An. coustani*	5	0 (0.0)	1 (20.0)	3 (60.0)	0 (0.0)	1 (20.0)	0 (0.0)	0 (0.0)	0 (0.0)
Total	238	24 (10.0)	81 (33.9)	33 (13.8)	18 (7.5)	3 (1.3)	4 (1.7)	1 (0.4)	75 (31.4)

Values in table are given as the number (*n*) of blood-meal sources with the percentage given in parentheses

## Discussion

This study provides additional information on malaria transmission dynamics and elucidates the spatial–temporal variation, composition and diversity of malaria vectors, trophic preferences and plasmodial infection status in Taita-Taveta County, Kenya. Understanding the distribution, species composition, infectivity and trophic preference of mosquitoes is essential for designing targeted control measures. During the course of the present study seven different species of mosquitoes were collected in the different study areas at varying densities: *An. funestus*, *An. gambiae*, *An. coustani*, *An. maculipalpis*, *An. moucheti*, *An. pharoensis*, *An. pretoriensis* and *An. squamosus*. *Anopheles funestus*, *An. gambiae* and *An. coustani* were collected at relatively higher densities. *Anopheles funestus* and *An. gambiae* were collected in all of the study villages. The densities of *Anopheles* mosquitoes were compared during the wet season and dry season and found to be overall higher in the wet season, likely attributable to the increase in larval development sites during the rainy season. *Anopheles gambiae* s.l. was present at significantly high numbers during the wet season. Mosquito abundance and diversity could also have been modified by agronomic activities and seasonality in the study area. Unplanned agricultural activities involving irrigation systems may be a factor that could have propagated ideal larval developmental sites, establishing adult mosquitoes and hence malaria transmission at these sites [40–42]. Additionally, during the rainy season there is an upsurge in the number of aquatic sites able to support larval development, thereby increasing the abundance of malaria vectors. The diversity and abundance of ideal larval developmental sites for *Anopheles* positively impacts on the resulting adult mosquito population and, consequently, the dynamics of malaria transmission. Agricultural practices have also been shown to influence the diversity, abundance and distribution of mosquito species [40]. However, we observed the reverse for *An. funestus* s.l. In Kenya, *An. funestus* prefers breeding in large semi-permanent habitats along river streams that are characterized by vegetation and algae [43]. In Tanzania, *An. funestus* have been shown to oviposit in permanent and semi-permanent small springs, natural ponds and river tributaries with clear water covered with vegetation [44]. In Taita-Taveta County, the presence of river water used for irrigation creates semi-permanent water bodies that are ideal habitats for mosquito breeding. During the dry season, water levels fall, possibly leading to reduced mosquito breeding. These results clearly indicate that vectors will have ideal developmental sites year in and year out. In different eco-epidemiological regions of Africa, these mosquitoes prefer oviposition in savannah with higher exposure to sunlight and temperature [45, 46]. Such larval developmental sites remain productive for a longer time and in most cases, *An. funestus* density peaks during the dry season.

The study also adds to the body of information on the role of complemental vectors in malaria transmission. Sporozoite antigens were detected in *An. gambiae* and *An. funestus*, the main malaria vectors, as well as in *An. coustani*. This result corroborates the findings of a previous study conducted in the same area [10], which highlighted the role of *An. parensis* and *An. coustani* in malaria transmission. Evidence has also been generated on the role of secondary vectors in malaria transmission [17, 18]. The sporozoite rate in our study was high in the wet season compared to the dry season. This could be attributed to increased densities of malaria vectors, which in turn would increase overall vector-human contact and the likelihood of some vectors surviving the extrinsic incubation period, thereby transmitting malaria. The majority of these vectors prefer biting and resting outdoors. *Anopheles coustani* s.l. mosquitoes have been demonstrated to be relaxed or flexible feeders with a preference for the outdoor environment. In our study, they tested positive for* Plasmodium* antigens, indicating their role as a secondary vector in malaria transmission. Several secondary vectors have been described as contributing to malaria transmission when interventions only target and affect the main malaria vectors. *Anopheles coustani* have been consistently found to harbour *Plasmodium* antigen in the DRC, Tanzania and Kenya [10, 19, 47]. Moreover, a study conducted in Zambia demonstrated an unexpected anthropophily in *An. coustani* [48]. These behaviours could be naturally acquired or they could have been induced due to the use of indoor-based interventions. Human behaviours have been shown to be one of the factors contributing to outdoor malaria transmission. A recent study indicated that human behaviour associated with social and economic activities have in some way contributed to outdoor transmission [14]. Socio-cultural activities, such as burial ceremonies, weddings, leisure activities and rituals, are among the factors that keep people outdoors and unprotected [14, 49].

*Anopheles gambiae* s.l. collected in the indoor environment had the highest blood-feeding indices. Amongst the mosquitoes which had fed on a single host, feeding on humans was found to be low while the goat was the most preferred single host. *Anopheles funestus* and *An. coustani* were found to prefer feeding on goats while *An. gambiae* preferred feeding on bovines/cows. Although some mosquitoes were found to be having mixed-feeding behaviour, combination blood-meal sources were found at only a low frequency amongst the tested mosquitoes. The high number of mosquitoes collected indoors that had also fed on an animal could be due to humans and animals sharing the house because of security issues or due to mosquitoes finding refuge indoors after feeding outdoors. Our results provide additional information on the biting behaviours of members of *An. gambiae* s.l. Our trophic preference analysis revealed that the majority of *An. gambiae* s.l. were highly zoophillic as they obtained blood from bovine and goat. These finds are consistent with the results of previous studies [10, 14]. The HBI for *An. gambiae* s.l. was approximately 0.14, indicating that approximately 14% of the collected *An. gambiae* s.l. had obtained blood meals from a human source as compared to other vertebrate sources. On the other hand, none of the *An. funestus* s.l. had obtained blood from human hosts, indicating that the sibling species in circulation are composed of highly zoophillic mosquitoes, such as *An. parensis*, *An. rivulorum* and *An. leesoni*, that had obtained meals from goat and bovine sources. A similar trend was observed for *An. coustani*, which showed a preference for vertebrate sources other than humans, thus indicating the zoophillic nature of this species and its accidental feeding on humans and, consequently, its transmission of parasites. These observations strongly support the urgent need to develop new and complimentary strategies which target outdoor transmission that occurs when people are unprotected. Several strategies are in the pipeline to address outdoor malaria transmission, such as spatial repellents, attractive toxic sugar bait (ATSB), ivermectin and gene drive technology, amongst others which might complement current interventions [50–58].

The present study is characterized by several limitations. First, molecular detection of* Plasmodium* and species identification were performed several years after sample collection. The first field collection was conducted in November 2016, and the mosquito samples were collected between 2016 and 2018. The samples were processed for ELISA procedures, and subsequently the ELISA homogenates/lysates were preserved in freezers at − 20 °C until DNA extraction and subsequent re-evaluation/analysis by PCR in 2022. The DNA material in some of the homogenates/lysates of the mosquito samples could have degraded, which may have resulted in the high amplification rate seen in the assays on species identification and* Plasmodium* detection. The degradation of homogenates/lysates and DNA in our study was not unexpected. A similar challenge was faced in a study conducted in Cameroon in which human samples collected in 2015 were screened for the presence of* Plasmodium* 18S ribosomal RNA. The samples were then re-evaluated in 2017, and only a fraction (62%) of the samples that initially tested positive were detected to be positive in the second evaluation [59]. Analysis/evaluation and re-evaluation of freshly collected samples are highly encouraged. Other factors that could have resulted in discordant results between the ELISA for* Plasmodium* circumsporozoite protein and PCR assays for* Plasmodium* detection could be the presence of bovine parasites in the highly zoophillic *An. coustani* [38, 39]. The proportionately high false positivity rate reported this study should serve as a warning to public health entomologists when interpreting ELISA results. Confirming ELISA results by PCR or other highly complementary methods in strongly recommended.

Secondly, only a subset of *An. gambiae* s.l. (*n* = 580, approx. 11.0%) and *An. funestus* (*n* = 103, approx. 28.9%) was identified by molecular tools into sibling species. The currently used surveillance tools for entomological surveillance is hampered by their high cost. Molecular identification is expensive and requires more time from DNA extraction to gel electrophoresis. Because of the limitations mentioned, entomological surveillance of wild vector populations in our study was hampered, limiting the scope of the analysis to just subpopulations of malaria vectors, thus leading to overgeneralization of the data. Assessing the role of each mosquito species in malaria transmission requires the development of robust and cost-effective methods for entomological surveillance that will inform decision-making on the composition of malaria vector species and the role of these species in malaria transmission.

## Conclusion

This study provides evidence that malaria transmission is occurring in both the indoor and outdoor environment in Taita-Taveta county, coastal Kenya. *Anopheles gambiae*,* An. funestus* and *An. coustani* were determined to be the key malaria vectors, both indoors and outdoors, transmitting malaria in both indoor and outdoor settings. All of the vectors tested showed a higher propensity for bovine and goat blood. Only *An. gambiae* was found to have fed on humans as a single host. More research is required to investigate the role of secondary vectors in malaria transmission. Also, the development of new complimentary strategies addressing the spatial–temporal gaps left behind by the current interventions by targeting outdoor transmission is urgently needed to tackle the transmission that occurs when people are unprotected.

## Data Availability

The supporting data are under the custodianship of the KEMRI-Wellcome Trust Data Governance Committee and are accessible upon request addressed to that committee.

## Abbreviations

ABTS: 2,2ʹ-Azino-bis (3-ethylbenzothiazoline-6-sulfonic acid)
b/p/n: Bites per person per night
CDC: US Centers for Disease Control and Prevention
ELISA: Enzyme-linked immunosorbent assay
HBI: Human blood index
HBR: Human biting rate
LLINs: Long-lasting insecticide-treated nets
PBS: Phosphate-buffered saline
